# Hyperbaric oxygen therapy: a novel treatment option in complex regional pain syndrome and other chronic pain conditions

**DOI:** 10.4103/mgr.MEDGASRES-D-25-00076

**Published:** 2026-04-11

**Authors:** Michal Hájek, Dittmar Chmelař, Adriana Poláčková, Marta Hodrová, Martin Dokulil, Jakub Tlapák, Ondřej Jor

**Affiliations:** Centre of Hyperbaric Medicine, Ostrava City Hospital, Ostrava, Czech Republic; Institute of Laboratory Medicine, Institute of Microbiology, Faculty of Medicine, University of Ostrava, Ostrava, Czech Republic; Centre for Hyperbaric Medicine of Faculty of Medicine University of Ostrava and Ostrava City Hospital, Ostrava, Czech Republic; Department of Anaesthesiology, Resuscitation and Intensive Care Medicine, Faculty of Medicine, University of Ostrava, Ostrava, Czech Republic; The Institute of Aviation Medicine, Prague, Czech Republic; Faculty of Biomedical Engineering, Czech Technical University in Prague, Czech Republic

A wide spectrum of conditions, including neuropathic pain, complex regional pain syndrome (CRPS), migraines, and fibromyalgia, are well-recognized contributors to chronic pain. CRPS is a chronic pain disorder characterized by spontaneous and evoked regional pain that is disproportionate to the expected path of pain after a similar trauma. This article explores the current understanding of CRPS pathophysiology, diagnosis, and treatment, while also examining future perspectives and emerging therapeutic strategies.

**Clinical manifestations and pathophysiological mechanisms:** CRPS manifests itself not only as pain, but also through motor and sensory alterations, including muscle atrophy, reduced mobility, edema, and changes in skin color in the affected region. The underlying pathophysiological mechanisms include musculoskeletal dysfunction, peripheral and central sensitization, autonomic dysregulation, sympathoafferent coupling, changes in the receptor population (e.g., up-regulation of the adrenergic receptor and decreased cutaneous nerve fibers), neuroplastic alterations, genetic predispositions, psychological factors, inflammation, and immune-mediated systemic and regional sympathetic disturbances.^1^

**Autophagy in the pathophysiology of chronic migraine and pain disorders:** Autophagy is a genetically regulated process in eukaryotic cells that mediates the degradation of cytoplasmic proteins and dysfunctional organelles through the lysosomal pathway, thereby preserving cellular homeostasis. The interactions among neurons, microglia, and astrocytes are critically involved in the pathophysiology of migraine. Nevertheless, the therapeutic implications of modulating autophagy for the prevention and management of migraine have not been adequately investigated. Emerging evidence has highlighted hyperbaric oxygen therapy (HBOT) as a potential intervention influencing autophagy. Based on preliminary research, HBOT may regulate mitophagy and relieve pain by mediating Ca^2+^/calmodulin-dependent protein kinase kinase beta/AMP-activated protein kinase signaling pathway in the neuropatic pain model. This autophagy-related intervention has yielded notable clinical benefits, and further research into the potential applications of autophagy modulators in the context of migraine and neuropathic pain management.^2^

**Diagnosis and biomarker development:** The diagnosis of CRPS is based primarily on clinical presentation and patient history, as no definitive laboratory or imaging modality provides absolute diagnostic confirmation. Although imaging techniques such as radiography, computed tomography, thermography, laser Doppler flowmetry, bone scintigraphy, magnetic resonance imaging, and electromyography can help in clinical assessment, none offer definitive diagnostic certainty. The research in CRPS aims to improve diagnostic precision through the identification of proteins and genetic markers associated with neuroinflammation. Although the likelihood of identifying a single CRPS biomarker remains low, a combination of multiple biomarkers can improve diagnostic accuracy and prognostication. Emerging research on noncoding microRNA signatures in blood samples holds promise for predicting treatment response and differentiating between acute and chronic CRPS; however, technological and economic constraints currently limit widespread clinical implementation.^3^

**Therapeutic approaches and multidisciplinary strategies:** The management of CRPS and chronic pain requires a multidisciplinary approach. Optimal results are achieved through comprehensive treatment regimens that integrate medical, psychological, physical, and occupational therapy interventions. Systematic reviews highlight a paucity of high-quality evidence supporting specific CRPS treatments, with limited data indicating the potential benefits of bisphosphonates, calcitonin, ketamine, graded motor imagery, and mirror therapy. Current treatment strategies prioritize physical therapy and occupational therapy to improve sensory perception, motor function, and sensorimotor integration.^4^ Psychological interventions targeting anxiety and maladaptive avoidance behaviors, pharmacological approaches that address pain and inflammation, passive physical therapies that aim to reduce edema and discomfort, and assistive devices to improve functional independence complement these strategies. Emerging therapeutic modalities include nerve blocks, neurostimulation techniques, and regenerative medicine approaches such as stem cell therapy.^4^

Repetitive transcranial magnetic stimulation has been investigated as a potential treatment for neuropathic pain conditions, including CRPS. A recent study evaluated the feasibility of repetitive transcranial magnetic stimulation for CRPS patients to reduce pain intensity and improve quality of life. Exploratory analysis revealed a significant reduction in pain intensity immediately following treatment.^5^ The combined application of HBOT and TMS has demonstrated promising therapeutic outcomes in patients suffering from chronic migraine, spinal cord injury, and cerebral infarction.^6^,^7^ Early intensive rehabilitation training combined with HBOT has been shown to confer greater benefits in the recovery of cognitive function, activities of daily living, and motor abilities in patients with traumatic brain injury.^8^ In patients diagnosed with CRPS, current pharmacological regimens, and physiotherapeutic approaches tend to follow a broadly similar therapeutic pattern. Data from a recent study shown that at least 68% of patients received HBOT in conjunction with calcium and vitamin D3 supplementation, either individually or in combination. Approximately 20% of patients were treated with antidepressants or anxiolytics, 20% received nonsteroidal anti-inflammatory drugs, and another 20% were administered analgesics. Additionally, 14% of patients were treated with antiepileptic agents possessing analgesic properties, such as pregabalin or gabapentin. Regarding rehabilitative interventions, 65% of patients underwent HBOT combined with comprehensive rehabilitation programs, while 29% received individualized physiotherapy, among other modalities.^9^

**Biological and neuromodulatory effects of hyperbaric oxygen therapy:** The persistent need for effective and well-tolerated therapies for chronic pain disorders has driven interest in HBOT as a noninvasive intervention with sustained efficacy and minimal adverse effects. The potential therapeutic benefits of HBOT in CRPS can be attributed to its modulation of hypoxia, acidosis, nitric oxide activity, and oxidative stress. HBOT has been shown to promote neuroplasticity, restore impaired brain function, and improve quality of life in patients recovering from stroke and traumatic brain injury. Its anti-inflammatory effects, mediated by the attenuation of pro-inflammatory glial mediators, further support its therapeutic potential in chronic pain conditions. In patients with fibromyalgia, HBOT has been reported to alter cerebral metabolism and glial function, potentially normalizing aberrant neural activity associated with the disorder.^10^

HBOT has been shown to significantly ameliorate mitochondrial abnormalities, optimize mitochondrial metabolism, improve compromised mitochondrial membrane integrity, and inhibit secondary cell death. This process is facilitated by mitochondrial transfer from astrocytes to neurons.^11^ After application of HBOT, adenosine triphosphate (ATP) levels, measured by enzyme-linked immunosorbent assay, showed a substantial increase in ATP expression, along with a higher NAD^+^ expression, a key marker of energy metabolism. A concurrent decrease in p53 and nuclear factor kappa B expression confirms that HBOT activates the ATP/NAD^+^/Sirit1 pathway. The activation of this pathway through HBOT has been associated with reduced cell necrosis and improved neurological function. The primary mitochondrial processes influenced by HBOT include induction of oxidative stress to stimulate adaptive antioxidant responses, enhancement of cellular energy metabolism through increased ATP and NAD^+^ production, and inhibition of apoptosis through modulation of key signaling pathways, particularly ATP/NAD^+^/Sirit1.^12^

**Figure 1:**
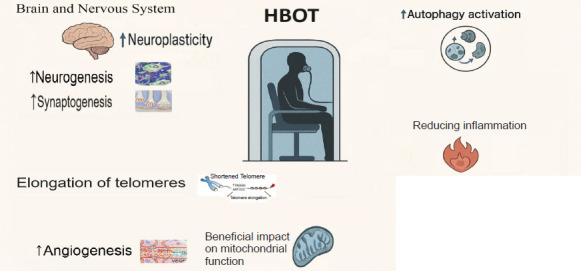
Biological and neuromodulatory effects of hyperbaric oxygen therapy (HBOT). Modified by Hájek M, based on OpenAI ChatGPT, 2025.

Although current literature provides compelling evidence for the neuroplastic effects of HBOT, a more comprehensive understanding of its underlying mechanisms may facilitate the development of more targeted and effective therapeutic strategies for neurological and neuropsychiatric disorders. According to a recent review, the neuromodulatory effects of HBOT include, in addition to the aforementioned enhancement of mitochondrial function and neuroprotection, the promotion of both neurogenesis and angiogenesis, facilitation of synaptic and axonal development, activation of the p38 mitogen-activated protein kinase signaling pathway, elongation of telomeres, anti-inflammatory actions, and the induction of brain-derived neurotrophic factor release.^13^ These findings have been corroborated by numerous clinical trials demonstrating significant improvements in cognitive function outcomes following HBOT in individuals with chronic traumatic brain injury, stroke, post-traumatic stress disorder, persistent post-concussion syndrome, fibromyalgia—as a representative condition of central sensitization syndrome, individuals with a history of childhood sexual abuse, as well as patients experiencing post-COVID-19 cognitive, psychiatric, fatigue, sleep, and pain symptoms, major depressive disorder and others. Clinically, these neuromodulatory effects of HBOT are reflected in enhanced recovery, improved cognitive functioning, and overall better quality of life among patients suffering from persistent neurological and psychiatric conditions.^13^ Biological and neuromodulatory effects of HBOT are illustrated in **Figure 1**.

**Clinical evidence supporting hyperbaric oxygen therapy for complex regional pain syndrome:** Over the past two decades, limited research has been published evaluating the efficacy of CRPS management. Only six case reports and one randomized controlled trial have documented its therapeutic effects in CRPS patients.^14^ Despite its potential, HBOT remains underrepresented in systematic reviews and meta-analyses, often omitted or excluded due to its nonstandardized role in CRPS treatment.

In a double-blind, randomized, placebo-controlled trial, 71 patients with post-traumatic CRPS of the upper extremity were enrolled, 37 were assigned to the HBOT group and 34 to the control group (receiving hyperbaric air). Both groups underwent 15 sessions of the hyperbaric chamber at 2.4 atmospheres absolute (ATA) for 90 minutes per session. Compared to the control group, patients treated with HBOT demonstrated statistically significant improvements (*P* < 0.001) in all parameters assessed, including pain reduction, swelling, and wrist flexion at 15 and 45 days post-treatment, except for wrist extension, which showed improvement but did not reach statistical significance.^14^

A recent retrospective study conducted in the Czech Republic analyzed 83 patients with CRPS who underwent HBOT at 2.0–2.4 ATA.^10^ The mean number of HBOT sessions was 22.0 ± 7.1. A total of 86% of patients reported symptomatic improvement following HBOT. Mean pain scores (Visual Analogue Scale) at rest decreased from 3.2 ± 3.0 to 1.6 ± 1.9 (*P* < 0.001), while pain scores during activity decreased from 6.1 ± 2.4 to 3.7 ± 2.4 (*P* < 0.001). The functional assessment of the affected limb improved from 7.0 ± 2.0 to 4.3 ± 2.4 (*P* < 0.001). In the final analysis of the 79 cases, 29% of patients exhibited a large clinically significant response, while 61% demonstrated a partial response with a minimally important difference. Further statistical analysis suggested that patients with a disease duration of less than three to six months exhibited a significantly higher response to HBOT (*P* = 0.029).^9^

A recent systematic review aimed to evaluate the efficacy of HBOT in the management of CRPS, focusing on the sympathetically maintained pain and sympathetically independent pain subtypes.^15^ The review included randomized controlled trials, retrospective observational studies, comparative studies, retrospective case series, and case reports published in English between January 1994 and October 2024. A total of 13 studies comprising 280 subjects were included in the analysis: 42.5% were classified as sympathetically maintained pain, 48.2% as sympathetically independent pain, and 9.3% as indeterminate. HBOT treatments ranged from 3 to 63 sessions, typically at 2.4 ATA for 90 minutes per session. The findings demonstrated that HBOT was effective in all CRPS subtypes: 97% of sympathetically maintained pain subjects, 94% of sympathetically independent pain subjects, and 100% of indeterminate subjects reported relief following HBOT. These results highlight significant symptom relief and functional improvements, suggesting HBOT as a valuable adjunct therapy in the management of CRPS.^15^

To conclude, CRPS remains a complex and debilitating medical condition that significantly impacts quality of life of patients. Current evidence on the effectiveness of HBOT in CRPS patients demonstrates a significant clinical impact on pain relief and enhancement of functional condition of the affected limb. The management of CRPS in the future will rely on technological innovations, regenerative medicine, and tailored treatment strategies, providing new therapeutic options for patients impacted by the condition.
